# Epigenetic modification of the Epstein-Barr virus BZLF1 promoter regulates viral reactivation from latency

**DOI:** 10.3389/fgene.2013.00053

**Published:** 2013-04-09

**Authors:** Takayuki Murata, Tatsuya Tsurumi

**Affiliations:** Division of Virology, Aichi Cancer Center Research InstituteNagoya, Japan

**Keywords:** epigenetics, Epstein–Barr virus, reactivation, latency, BZLF1 gene

## Abstract

The Epstein–Barr virus (EBV) is an oncogenic human gamma-herpesvirus that predominantly establishes latent infection in B lymphocytes. Viral genomes exist as extrachromosomal episomes with a nucleosomal structure. Maintenance of virus latency or execution of reactivation is controlled by the expression of BZLF1, a viral immediate-early gene product, tightly controlled at the transcriptional level. In this article, we review how BZLF1 transcription is controlled, in other words how virus reactivation is regulated, especially in terms of epigenetics. We recently found that histone H3 lysine 27 trimethylation (H3K27me3) and H4K20me3 markers are crucial for suppression of BZLF1 in latent Raji cells. In addition, H3K9me2/3, heterochromatin protein 1, and H2A ubiquitination are associated with latency, whereas positive markers, such as higher histone acetylation and H3K4me3, are concomitant with reactivation. Since lytic replication eventually causes cell cycle arrest and cell death, development of oncolytic therapy for EBV-positive cancers is conceivable using epigenetic disruptors. In addition, we note the difficulties in analyzing roles of epigenetics in EBV, including issues like cell type dependence and virus copy numbers.

## INTRODUCTION

The Epstein–Barr virus (EBV), a human gamma-herpesvirus that predominantly establishes latent infection in B lymphocytes, is associated with various disease entities, including Burkitt’s lymphoma, post-transplant lymphoproliferative disorder (PTLD), Hodgkin’s disease, gastric cancer, and nasopharyngeal carcinoma (NPC). Only a small percentage of infected cells switch from the latent stage into the lytic cycle and produce progeny viruses. Transitions and differences in EBV infection cycling between lytic and latent states are closely tied, not only with the virus production and spread, but also with disease progression and malignancy of EBV-positive cancers, and thus detailed analysis of molecular mechanisms that govern the EBV latent-to-lytic switch is of fundamental importance.

## LATENCY AND REACTIVATION OF EBV IS REGULATED BY AN ABUNDANCE OF BZLF1

**FIGURE 1:**
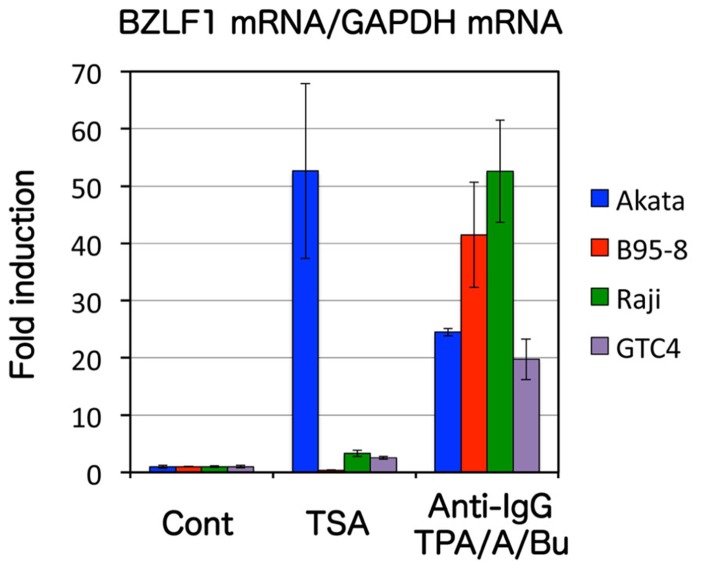
**Effects of an HDAC inhibitorTSA on BZLF1 expression differ with the type of EBV-positive cell.** Akata, Raji, B95-8, and GTC-4 cells were treated with either vehicle (Cont) or 300 nMTSA. Anti-IgG (for Akata) or TPA/A23187/butyrate (for B95-8, Raji, and GTC-4) served as positive controls, as these substances induce BZLF1. After 24 h, RNAs were collected and real-time RT-PCR was carried out to measure the levels of BZLF1 mRNA, the results being shown as bars after normalization to levels of glyceraldehyde 3-phosphate dehydrogenase (GAPDH) mRNA. TSA alone treatment induced BZLF1 expression in Akata, but did not appreciably enhance in other cells.

Although the mechanism of EBV reactivation *in vivo* is not fully understood, it is known to be elicited *in vitro* by treatment of latently infected B cells with some chemical or biological reagents, such as 12-*O*-tetradecanoylphorbol-13-acetate (TPA), calcium ionophores, sodium butyrate, and anti-immunoglobulin (Ig). Stimulation of the EBV lytic cascade by these reagents leads to expression of two viral immediate-early genes, BZLF1 (also known as Zta, EB1, ZEBRA, or Z) and BRLF1 (Rta or R). The BZLF1 protein is a transcriptional activator that shares structural similarities to basic leucine zipper (b-Zip) family transcriptional factors and BZLF1 expression alone can trigger the entire reactivation cascade (Speck et al., 1997; Amon and Farrell, 2005; Tsurumi et al., 2005). BZLF1 has a very interesting and unique characteristic trial. In cells latently infected with EBV, the viral lytic promoters are strongly repressed by repressive epigenetic marks, including heavy 5′-CG-3′ dinucleotide (CpG) DNA methylation (Fernandez et al., 2009), but BZLF1 can preferentially bind to and activate the methylated promoters (Bhende et al., 2004; Dickerson et al., 2009; Flower et al., 2011). Therefore, BZLF1 serves as the molecular switch for EBV reactivation from latency. Actually, induction of BZLF1 (20- to 50-folds) by anti-IgG or other chemical inducers (see **Figure 1**) can cause efficient viral gene expression, viral DNA replication and progeny production, at least in Akata or B95-8.

## POSITIVE/NEGATIVE CONTROL OF BZLF1 EXPRESSION BY TRANSCRIPTION FACTORS

Expression of the BZLF1 gene is tightly controlled at the transcriptional level. The BZLF1 promoter (Zp) normally exhibits low basal activity and is activated in response to TPA or the other reagents listed above. The promoter is activated by transcriptional factors including myocyte enhancer factor 2 (MEF2; Liu et al., 1997b) and Sp1/3 (Liu et al., 1997a). Cellular b-Zip type transcription factors, such as the cyclic AMP-response element-binding protein (CREB), activating transcription factor (ATF), activator protein-1 (AP-1; Ruf and Rawlins, 1995; Liu et al., 1998; Murata et al., 2009, 2011) or a spliced form of the X-box binding protein 1 [XBP-1(s); Bhende et al., 2007], also play crucial roles in the promoter activation. We previously showed the importance of CREB and its calcineurin-dependent activation by transducer of regulated CREB 2 (TORC2; Murata et al., 2009). Once produced, BZLF1 itself can bind to and activate its own promoter (Flemington and Speck, 1990; Murata et al., 2010). Most of the positive factors have been demonstrated or are presumed to up-regulate the BZLF1 promoter by recruiting transcriptional coactivators, such as histone acetylases. On the other hand, the activity of Zp is restricted by repressive factors including Jun dimerization protein 2 (JDP2; Murata et al., 2011), zinc finger E-box binding factor (ZEB; Yu et al., 2007), Yin Yang 1 (YY1; Montalvo et al., 1995), and an unidentified repressor that binds to the ZIIR motif (Liu et al., 1998).

## EPIGENETICS OF THE BZLF1 PROMOTER ASSOCIATED WITH LATENCY AND REACTIVATION

In the previous section, we noted that BZLF1 promoter activity is regulated positively or negatively by transcription factors and cofactors. The question then arises of how those host transcription factors regulate BZLF1 transcription, which eventually leads to EBV reactivation? The answer is through epigenetic changes that mediate transcription factors and BZLF1 expression.

With regard to epigenetics, CpG DNA methylation could be one possible cause of BZLF1 promoter repression, as this is frequently associated with constitutive heterochromatin, where transcription is tightly suppressed, irreversibly. However, Fernandez et al. (2009) showed that CpG methylation levels at BZLF1 promoters in various EBV-positive cell lines are exceptionally low, although most of the viral genome in the latent phase is highly methylated. Likewise, very little CpG DNA methylation was found in the promoter region of the lytic switch gene, ORF50/K-Rta, for Kaposi sarcoma-associated herpesvirus (KSHV), another oncogenic gamma-herpesvirus (Gunther and Grundhoff, 2010).

However, treatment of EBV-positive cells with 5-aza-2′-deoxycytidine (5-Aza), a potent inhibitor of DNA methyltransferase, induces BZLF1 transcription (Murata et al., 2012; see **Figure 3**). It is speculated that 5-Aza activates EBV lytic gene expression by an unknown mechanism that does not involve decreased CpG DNA methylation levels (Countryman et al., 2008). This is because 5-Aza induces BZLF1 expression within a very short period of time (15 min or less), although it must take days to bring about hypo-methylation of the CpG DNA, since 5-Aza is a DNA methyltransferase inhibitor and it does not actively trim off nor abolish methylation without *de novo* DNA amplification. In fact, accumulating data indicate that the mechanism of gene inductions by 5-Aza or its analogs is very complicated, and does not necessarily depend on DNA demethylation. The inhibitors can activate gene expressions through DNA damage (Link et al., 2008; Wang et al., 2008), degradation of a certain proteins (Zheng et al., 2012), or histone reorganization (Wozniak et al., 2007; Komashko and Farnham, 2010). Therefore, it is quite likely that the consequence of 5-Aza is a side effect, although the possibility cannot be denied that DNA methylation is present at Zp at least to some extent, and plays a role in BZLF1 gene suppression (Li et al., 2012).

Possible epigenetic modifications which might silence the promoter include histone changes. From a historical perspective, the best-characterized epigenetic histone marker of BZLF1 promoter is acetylation. Histone acetylation causes destabilization of chromatin, leading to a loose, open structure of the promoter, so that it becomes easily accessible to basic transcription factors. Histone acetylation of EBV Zp first came to light because histone deacetylase (HDAC) inhibitors were found to cause reactivation of EBV (Luka et al., 1979; Jenkins et al., 2000). Histone acetylation levels are low in latency, and are induced upon reactivation (Murata et al., 2012). In fact, silencing of the BZLF1 promoter in latently infected cells is mediated by and solely dependent on low levels of histone acetylation, at least in some cell lines such as Akata, since inhibitors of HDAC, like sodium butyrate or trichostatin A (TSA), can reverse the silencing (Miller et al., 2007; Murata et al., 2012; **Figure 1**). However, treatment with butyrate or TSA alone does not efficiently induce BZLF1 transcription in cell lines like B95-8 or Raji, suggesting that the molecular mechanisms that govern the suppression of BZLF1 transcription in these cells must be more complex than simply reduction in the acetylation level of the promoter (Countryman et al., 2008; Murata et al., 2012; **Figure 1**).

**FIGURE 2:**
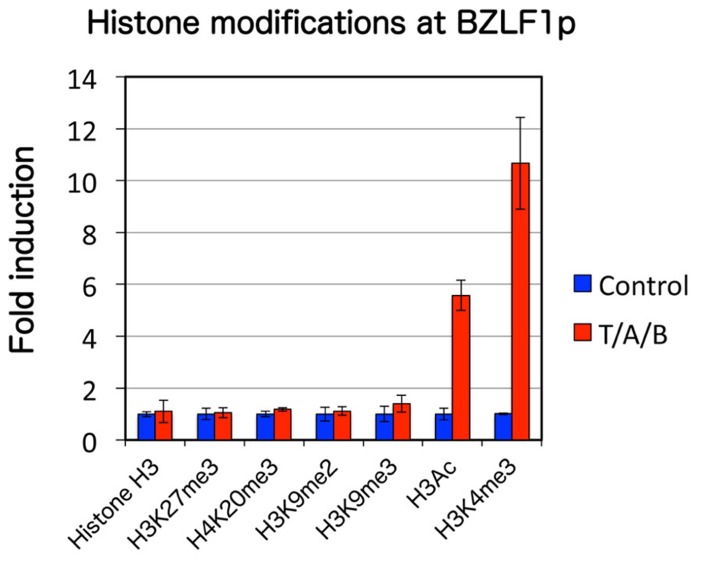
**Histone modification pattern of EBV BZLF1 promoter upon lytic reactivation.** Raji cells were treated with control vehicle (blue) or TPA/A23187/butyrate (T/A/B, red) at 20 ng/ml, 1 μM and 5 mM, respectively. ChIP experiments were performed using antibodies indicated below, followed by real-time PCR. Active marks (H3Ac, H3K4me3) were elevated while levels of suppressive marks (H3K27me3, H4K20me3, and H3K9me2/3) remained unchanged.

In order to analyze mechanisms that govern BZLF1 transcription other than histone acetylation in such a cell line, we first examined various epigenetic histone modifications in the Zp of EBV DNA. Chromatin immunoprecipitation (ChIP) assays revealed that suppressive histone markers including histone H3 lysine 27 trimethylation (H3K27me3), H3K9me2/3 and H4K20me3 are present in the Zp of latent Raji cells, while high levels of histone acetylation and H3K4me3 markers correlate with reactivation of the virus (**Figure 2**; Murata et al., 2012).

**FIGURE 3:**
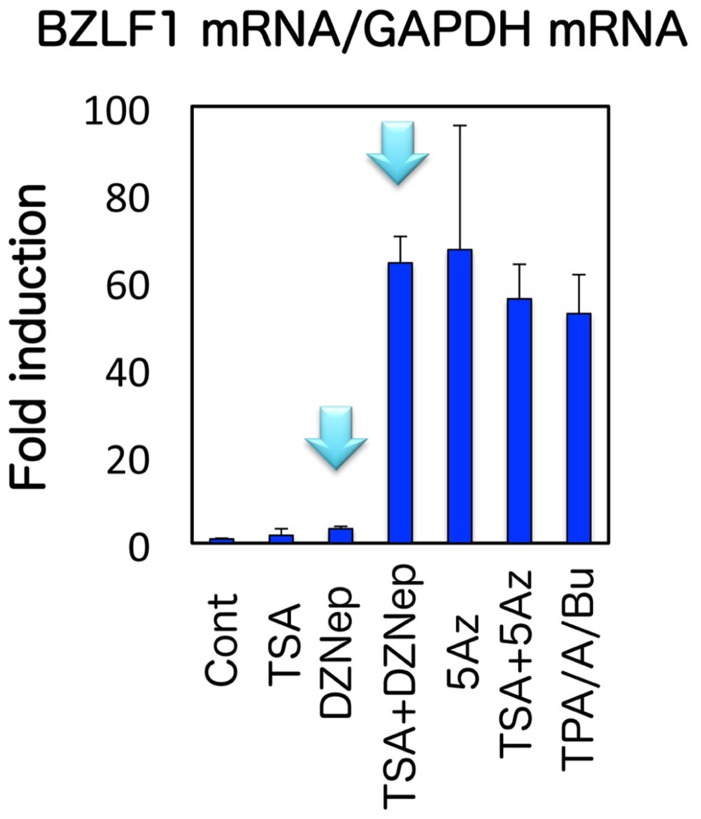
**Effects of pharmacological inhibitors on BZLF1 expression in Raji cells.** Raji cells were treated with vehicle (Cont), 10 μM DZNep, 300 nMTSA, 1 μM 5-Aza alone or in combinations as indicated. As a positive control (TPA/A/Bu), Raji cells were treated with TPA/A23187/butyrate at 20 ng/ml, 1 μM and 5 mM, respectively. For DZNep or 5-aza-2′-deoxycytidine (5-Aza) treatment, cells were exposed to the reagent daily for 3 days. Treatment with other chemicals was for 24 h. Real-time RT-PCR was carried out to measure the levels of BZLF1 mRNA, which were then normalized to GAPDH mRNA levels. The light-blue arrows indicate that DZNep alone did not efficiently induce BZLF1, but could markedly enhance the expression if treated in combination with TSA.

H3K27me3 is a suppressive histone modification, characteristic of facultative heterochromatin, a form of heterochromatin where expression of a wide variety of genes is considerably silenced by specific histone modifications (Kondo, 2009). With specific signaling, histone modifications of this type of heterochromatin can be reversed so that it becomes transcriptionally active, unlike constitutive heterochromatin. The presence of H3K27me3 methylation was recently reported by other groups in EBV Zp (Ramasubramanyan et al., 2012) and KSHV ORF50/K-Rta (Gunther and Grundhoff, 2010; Toth et al., 2010). To test if H3K27me3 modification is involved in the BZLF1 suppression during latency, we here used an inhibitor of the modification, 3-deazaneplanocin A (DZNep; Tan et al., 2007; Miranda et al., 2009). While treatment of Raji cells with either DZNep or TSA alone had only minor effects on BZLF1 levels (1.8- and 3.3-fold increase, respectively), use of the two inhibitors in combination (TSA + DZNep) stimulated the expression 64.2-fold (Murata et al., 2012; **Figure 3**). This result suggests that not only histone deacetylation but also histone H3K27me3 serve to inhibit BZLF1 transcription, at least in Raji cells. H3K27me3 methylation is mediated by enhancer of zeste 2 (Ezh2), a member of polycomb repressor complex 2 (PRC2; Cao et al., 2002). To further verify the involvement of H3K27me3 in BZLF1 gene repression, we then knocked down Ezh2. Silencing increased BZLF1 levels by 2.5-fold even without TSA, and addition of TSA elevated this to 10.9-fold (Murata et al., 2012). Furthermore, we confirmed these inhibitors and small interfering RNA (siRNA) treatment actually caused expected changes in epigenetic marks (see Figures 7 and 9 in Murata et al., 2012). An importance of histone H3K27me3 in the maintenance of latency was also recently demonstrated for KSHV ORF50/K-Rta (Toth et al., 2010). These results point to involvement of Ezh2 methyltransferase and the histone H3K27me3 marker in silencing of BZLF1 gene expression during EBV latency. In addition, we would like to note that histone acetylation is also needed for efficient expression of BZLF1.

It has been reported that, in addition to histone H3K27me3, H4K20me3 histone modification is also inhibited by DZNep (Tan et al., 2007; Miranda et al., 2009), and we found H4K20me3 is present in the Zp of latent Raji cells. In order to specifically examine the effect of the H4K20me3 methylation on silencing of the BZLF1 gene, Suv420h1, the methyltransferase responsible for the modification, was knocked down by siRNA technology. Remarkable induction of the BZLF1 gene by Suv420h1 knockdown and TSA corresponded with reduction of H4K20me3 levels and elevation of active H3K9Ac and H3K4me3 markers (see Figures 7 and 10 in Murata et al., 2012). Therefore, we conclude that silencing of the BZLF1 promoter in Raji cells is similarly brought about by histone H4K20me3 methylation.

Because DZNep exhibited potent inducing effects on BZLF1 gene transcription, we also tested BIX01294, a specific inhibitor of G9a, the methyltransferase responsible for histone H3K9me2 methylation, which is another typical marker of facultative heterochromatin. Paradoxically, treatment of Raji cells or other EBV-positive cells with BIX01294 alone or in combination with TSA, DZNep, or 5-Aza, did not increase the BZLF1 expression at all, or caused very modest increase at most, even though H3K9me2 is present at the Zp at significantly high level (Murata et al., 2012). The data imply that K3K9me2 may not play an important role in the suppression of BZLF1, at least in Raji cells.

A representative constitutive heterochromatin marker histone H3K9me3, too, has been reported to be definitely present during latency in EBV Zp (Murata et al., 2012) and KSHV ORF50/K-Rta promoters (Gunther and Grundhoff, 2010; Toth et al., 2010). Although Toth and others observed that H3K9me3 in the KSHV ORF50/K-Rta promoter decreased upon induction, suggesting that the modification is involved in silencing of the immediate-early gene, we failed to see an equivalent decline in EBV Zp (Murata et al., 2012; **Figure 2**). We speculate that this inconsistency was related to the use of Raji cells in our experiments, since the Raji genome has a deletion of the BALF2 gene, essential for lytic viral DNA synthesis. Furthermore, treatment of latent Raji cells with chaetocin, an Suv39H1 histone H3K9me3 methyltransferase inhibitor, did not induce BZLF1 expression, even in combination with other epigenetic inhibitors, such as TSA, DZNep, or 5-Aza. Therefore, histone H3K9me3 modification is a feature of EBV Zp in latency, but we still do not have conclusive evidence that it plays a role in the maintenance of latency, at least in Raji cells. Since other methyltransferases, such as SETDB1/ESET, can also catalyze histone H3K9me3 modification, they may be acting to suppress BZLF1 gene in the presence of chaetocin.

Histone H3K4me3 is enriched in the promoter regions of transcriptionally active genes in euchromatin, and thus serves as an active chromatin marker. It elicits transcription by recruiting factors like chromodomain-containing and plant homeodomain (PHD) finger proteins, as well as chromatin remodeling factors. Lytic induction of Raji cells markedly elevated the active histone marker, H3K4me3, in the Zp, while the level was low in latency (Murata et al., 2012; **Figure 2**). Enhancement of H3K4me3 upon induction has been reported for KSHV (Gunther and Grundhoff, 2010; Toth et al., 2010) in addition to other herpesviruses, indicating that histone H3K4me3 methylation, like histone acetylation, plays an important and universal role in lytic gene expression of herpesviruses.

Further to the epigenetic modifications described above, already published by us or other groups, we have confirmed in our preliminary experiments that other epigenetic alterations are associated with EBV latency and reactivation. In the mammalian genome, approximately 10% of histone H2A is mono-ubiquitinated at Lys 119, in association with transcriptional suppression, and then de-ubiquitinated upon transcriptional activation. We have found EBV Zp of latent Raji cells to be labeled with high levels of mono-ubiquitinated H2A, although massive reduction did not occur on induction. A similar ChIP result was obtained when heterochromatin protein 1 (HP1) was monitored. Because HP1 binds to methylated histone H3K9, the presence of HP1 serves to strengthen significance of H3K9 methylation at the promoter. Thus, BZLF1 promoter has various repressive epigenetic modifications, and also acquires cofactors associated for the gene suppression. Physiological relevance of those factors is being analyzed.

Interestingly, we found there is a prominent binding site of CCCTC-binding factor (CTCF), a transcriptional regulator and insulator, in the Zp of EBV. Binding of CTCF correlates with binding of Rad21, a subunit of cohesion. Other groups also recently confirmed such binding to EBV Zp (Holdorf et al., 2011; Arvey et al., 2012). Since it is known that CTCF/cohesin regulate transcription by creating long range chromatin loops and/or by acting as insulators, roles of such factor binding in latency and reactivation of EBV is of great interest. Binding of CTCF/cohesin to the KSHV ORF50/K-Rta promoter and a contribution to the suppression of reactivation have already been established (Chen et al., 2012), but their role in EBV reactivation may be different, as the binding sites of CTCF/cohesin in the ORF50/K-Rta promoter appear redundant while there is only one major peak of CTCF/cohesin in the EBV BZLF1 promoter (Holdorf et al., 2011; Arvey et al., 2012). We recently made recombinant EBV with point mutation at the CTCF binding site of the BZLF1 promoter, but our preliminary data showed that disruption of CTCF binding to the peak did not notably influence on BZLF1 levels, if any, at least in HEK293 cells.

## EPIGENETIC AGENTS AS MOLECULAR TARGETS FOR ANTI-VIRAL/CANCER DRUGS

Because there are very limited numbers of anti-EBV drugs developed or being developed to date, including acyclic nucleoside analogs, such as acyclovir or ganciclovir, and kinase inhibitors, such as maribavir (Wang et al., 2009), the search and development of effective anti-viral drugs for patients with infectious mononucleosis, caused by primary and acute EBV infection in adolescence, are important tasks. Because histone acetylation plays a crucial role in EBV reactivation, inhibitors of histone acetyl transferase (HAT) have potential in this regard. Inhibition of histone demethylase LSD1 by monoamine oxidase inhibitors is reported to block alpha herpesvirus lytic replication and reactivation from latency (Liang et al., 2009).

Interestingly, as execution of the viral lytic program arrests cell cycle progression in infected cells (Kudoh et al., 2003), induction of EBV lytic replication in EBV-positive cancers by epigenetic inhibitors, such as HDAC inhibitors, 5-Aza, and/or DZNep, may offer clinical application as a type of oncolytic therapy in the future (Feng et al., 2004; Jung et al., 2007). Because treatment like this must induce efficient production of progeny viruses, anti-viral drugs, such as ganciclovir, should obviously be used in combination to both induce apoptosis and prevent viral spreading.

## PARTICULARITY AND DIFFICULTIES OF ANALYZING EBV EPIGENETICS

It must be emphasized that responses of BZLF1 promoter activity to certain epigenetic inhibitors depend largely on the cell type. To take one example, levels of BZLF1 mRNA expression in Akata cells are markedly induced by TSA treatment alone, whereas the virus in other cells, including B95-8 or Raji, does not appear to respond (Murata et al., 2012; **Figure 1**). We have demonstrated, in Raji cells, that BZLF1 expression is suppressed by histone H3K27me3 and H4K20me3, in addition to low level histone acetylation, whilst in Akata cells, only low level histone acetylation accounts for repression of the gene induction (Murata et al., 2012). Curiously however, the Zp of the Akata cell line, is modified with histone H3K27me3 and H4K20me3, almost as efficiently as Raji (Murata et al., 2012). Then, why do the suppressive H3K27me3 and H4K20me3 markers not actually prevent BZLF1 expression in Akata cells?

Another question is why treatment with TPA, A23187, and sodium butyrate did not affect repression markers, such as H3K9me2/3, H3K27me3, or H4K20me3, at all in Raji, whereas they significantly elicited expression of BZLF1 (Murata et al., 2012)? It is considered in general that such suppressive markers must be diminished for transcriptional activation.

We believe these inconsistencies can be explained in terms of latent EBV genome copy numbers. To take an example, it is known that about 5–100 copies of the episomal EBV genome are present per latent cell. For the first question, let us suppose there are 10 copies of the latent EBV genome in one Akata cell, and nine copies are modified and repressed by suppressive H3K27me3 and H4K20me3 markers, the remaining copy being unmodified. This means the virus in Akata cells retains high sensitivity to TSA alone. For the second question, if TPA/A23187/butyrate treatment of Raji cells induces reduction of such repressive histone methylation in only a few copies but still allows efficient expression of BZLF1, reduction of the repressive modification must be difficult to detect, because the histone methylations in the majority of the genome copies are intact. In contrast, induction of active histone markers, like histone acetylation or H3K4me3 methylation, can clearly be observed.

In addition, the presence of epigenetic markers, like H3K27me3 or H3K4me3, may not in itself be sufficient for suppression or activation. Adaptor or mediator complexes, such as polycomb-group proteins or PHD finger proteins must be recruited to the promoter regions and appropriately act to compact or open the chromatin structure. Therefore, we suggest that only presence or absence of a certain epigenetic alteration in any regulatory region of EBV does not necessarily mean that it is critical. For determination of actual significance, functional assays, such as use of specific inhibitors and knockdown of epigenetic enzymes, are essential.

## SUMMARY

**FIGURE 4:**
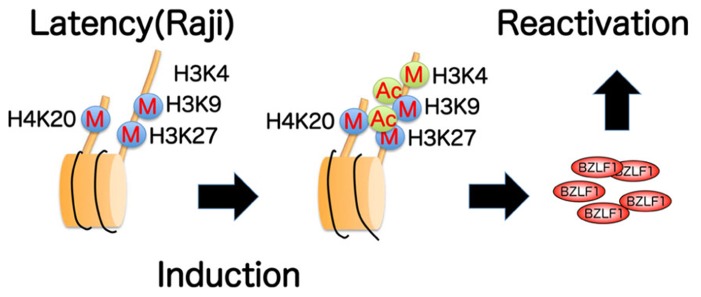
**Summary of epigenetic histone modifications in the BZLF1 promoter of Raji cells.** Repressive histone H3K9, H3K27, H4K20 methylations (marked with blue circles) are present in latency, and are not appreciably decreased even after induction. High levels of active markers, such as H3K4 methylation and histone acetylation (green circles), are notably associated with lytic induction.

We recently found (Murata et al., 2012) that histone H3K27me3 and H4K20me3 markers are crucial for maintenance of EBV latency, while histone acetylation and H3K4me3 are associated with reactivation from latency, at least in Raji cells (**Figure 4**). Although there may be differences in response between cell types, these data provide primary evidence for potential in anti-viral/cancer drug development.

## Conflict of Interest Statement

The authors declare that the research was conducted in the absence of any commercial or financial relationships that could be construed as a potential conflict of interest.

## References

[B1] Amon W. Farrell P. J. (2005). Reactivation of Epstein–Barr virus from latency. *Rev. Med. Virol.* 15 149–156 15546128 10.1002/rmv.456

[B2] Arvey A. Tempera I. Tsai K. Chen H. S. Tikhmyanova N. Klichinsky M. (2012). An atlas of the Epstein–Barr virus transcriptome and epigenome reveals host–virus regulatory interactions. *Cell Host Microbe* 12 233–245 22901543 10.1016/j.chom.2012.06.008 PMC3424516

[B3] Bhende P. M. Dickerson S. J. Sun X. Feng W. H. Kenney S. C. (2007). X-box-binding protein 1 activates lytic Epstein–Barr virus gene expression in combination with protein kinase D. *J. Virol.* 81 7363–7370 17494074 10.1128/JVI.00154-07 PMC1933364

[B4] Bhende P. M. Seaman W. T. Delecluse H. J. Kenney S. C. (2004). The EBV lytic switch protein, Z, preferentially binds to and activates the methylated viral genome. *Nat. Genet.* 36 1099–1104 15361873 10.1038/ng1424

[B5] Cao R. Wang L. Wang H. Xia L. Erdjument-Bromage H. Tempst P. (2002). Role of histone H3 lysine 27 methylation in polycomb-group silencing. *Science* 298 1039–1043 12351676 10.1126/science.1076997

[B6] Chen H. S. Wikramasinghe P. Showe L. Lieberman P. M. (2012). Cohesins repress Kapos’s sarcoma-associated herpesvirus immediate early gene transcription during latency. *J. Virol.* 86 9454–9464 22740398 10.1128/JVI.00787-12 PMC3416178

[B7] Countryman J. K. Gradoville L. Miller G. (2008). Histone hyperacetylation occurs on promoters of lytic cycle regulatory genes in Epstein–Barr virus-infected cell lines which are refractory to disruption of latency by histone deacetylase inhibitors. *J. Virol.* 82 4706–4719 18337569 10.1128/JVI.00116-08 PMC2346723

[B8] Dickerson S. J. Xing Y. Robinson A. R. Seaman W. T. Gruffat H. Kenney S. C. (2009). Methylation-dependent binding of the Epstein–Barr virus BZLF1 protein to viral promoters. *PLoS Pathog.* 5:e1000356 10.1371/journal.ppat.1000356 PMC2654727 19325883

[B9] Feng W. H. Hong G. Delecluse H. J. Kenney S. C. (2004). Lytic induction therapy for Epstein–Barr virus-positive B-cell lymphomas. *J. Virol.* 78 1893–1902 14747554 10.1128/JVI.78.4.1893-1902.2004 PMC369434

[B10] Fernandez A. F. Rosales C. Lopez-Nieva P. Grana O. Ballestar E. Ropero S. (2009). The dynamic DNA methylomes of double-stranded DNA viruses associated with human cancer. *Genome Res.* 19 438–451 19208682 10.1101/gr.083550.108 PMC2661803

[B11] Flemington E. Speck S. H. (1990). Autoregulation of Epstein–Barr virus putative lytic switch gene BZLF1. *J. Virol.* 64 1227–1232 2154606 10.1128/jvi.64.3.1227-1232.1990 PMC249237

[B12] Flower K. Thomas D. Heather J. Ramasubramanyan S. Jones S. Sinclair A. J. (2011). Epigenetic control of viral life-cycle by a DNA-methylation dependent transcription factor. *PLoS ONE* 6:e25922 10.1371/journal.pone.0025922 PMC3191170 22022468

[B13] Gunther T. Grundhoff A. (2010). The epigenetic landscape of latent Kaposi sarcoma-associated herpesvirus genomes. *PLoS Pathog.* 6:e1000935 10.1371/journal.ppat.1000935 PMC2880564 20532208

[B14] Holdorf M. M. Cooper S. B. Yamamoto K. R. Miranda J. J. (2011). Occupancy of chromatin organizers in the Epstein–Barr virus genome. *Virology* 415 1–5 21550623 10.1016/j.virol.2011.04.004 PMC3808970

[B15] Jenkins P. J. Binne U. K Farrell P. J. (2000). Histone acetylation and reactivation of Epstein–Barr virus from latency. *J. Virol.* 74 710–720 10623733 10.1128/jvi.74.2.710-720.2000 PMC111591

[B16] Jung E. J. Lee Y. M. Lee B. L. Chang M. S. Kim W. H. (2007). Lytic induction and apoptosis of Epstein–Barr virus-associated gastric cancer cell line with epigenetic modifiers and ganciclovir. *Cancer Lett.* 247 77–83 16647201 10.1016/j.canlet.2006.03.022

[B17] Komashko V. M. Farnham P. J. (2010). 5-azacytidine treatment reorganizes genomic histone modification patterns. *Epigenetics* 5 229–240 10.4161/epi.5.3.11409 20305384

[B18] Kondo Y. (2009). Epigenetic cross-talk between DNA methylation and histone modifications in human cancers. *Yonsei Med. J.* 50 455–463 19718392 10.3349/ymj.2009.50.4.455 PMC2730606

[B19] Kudoh A. Fujita M. Kiyono T. Kuzushima K. Sugaya Y. Izuta S. (2003). Reactivation of lytic replication from B cells latently infected with Epstein–Barr virus occurs with high S-phase cyclin-dependent kinase activity while inhibiting cellular DNA replication. *J. Virol.* 77 851–861 12502801 10.1128/JVI.77.2.851-861.2003 PMC140784

[B20] Li L. Su X. Choi G. C. Cao Y. Ambinder R. F. Tao Q. (2012). Methylation profiling of Epstein–Barr virus immediate-early gene promoters, BZLF1 and BRLF1 in tumors of epithelial, NK- and B-cell origins. *BMC Cancer* 12:125 10.1186/1471-2407-12-125 PMC3362778 22458933

[B21] Liang Y. Vogel J. L. Narayanan A. Peng H. Kristie T. M. (2009). Inhibition of the histone demethylase LSD1 blocks alpha-herpesvirus lytic replication and reactivation from latency. *Nat. Med.* 15 1312–1317 19855399 10.1038/nm.2051 PMC2783573

[B22] Link P. A. Baer M. R. James S. R. Jones D. A. Karpf A. R. (2008). p53-inducible ribonucleotide reductase (p53R2/RRM2B) is a DNA hypomethylation-independent decitabine gene target that correlates with clinical response in myelodysplastic syndrome/acute myelogenous leukemia. *Cancer Res.* 68 9358–9366 19010910 10.1158/0008-5472.CAN-08-1860 PMC2606040

[B23] Liu P. Liu S. Speck S. H. (1998). Identification of a negative *cis* element within the ZII domain of the Epstein–Barr virus lytic switch BZLF1 gene promoter. *J. Virol.* 72 8230–8239 9733866 10.1128/jvi.72.10.8230-8239.1998 PMC110177

[B24] Liu S. Borras A. M. Liu P. Suske G. Speck S. H. (1997a). Binding of the ubiquitous cellular transcription factors Sp1 and Sp3 to the ZI domains in the Epstein–Barr virus lytic switch BZLF1 gene promoter. *Virology* 228 11–18 9024805 10.1006/viro.1996.8371

[B25] Liu S. Liu P. Borras A. Chatila T. Speck S. H. (1997b). Cyclosporin A-sensitive induction of the Epstein–Barr virus lytic switch is mediated via a novel pathway involving a MEF2 family member. *EMBO J.* 16 143–153 9009275 10.1093/emboj/16.1.143 PMC1169621

[B26] Luka J. Kallin B. Klein G. (1979). Induction of the Epstein–Barr virus (EBV) cycle in latently infected cells by *n*-butyrate. *Virology* 94 228–231 220786 10.1016/0042-6822(79)90455-0

[B27] Miller G. El-Guindy A. Countryman J. Ye J. Gradoville L. (2007). Lytic cycle switches of oncogenic human gammaherpesviruses. *Adv. Cancer Res.* 97 81–109 17419942 10.1016/S0065-230X(06)97004-3

[B28] Miranda T. B. Cortez C. C. Yoo C. B. Liang G. Abe M. Kelly T. K. (2009). DZNep is a global histone methylation inhibitor that reactivates developmental genes not silenced by DNA methylation. *Mol. Cancer Ther.* 8 1579–1588 19509260 10.1158/1535-7163.MCT-09-0013 PMC3186068

[B29] Montalvo E. A. Cottam M. Hill S. Wang Y. J. (1995). YY1 binds to and regulates *cis*-acting negative elements in the Epstein–Barr virus BZLF1 promoter. *J. Virol.* 69 4158–4165 7769675 10.1128/jvi.69.7.4158-4165.1995 PMC189152

[B30] Murata T. Hotta N. Toyama S. Nakayama S. Chiba S. Isomura H. (2010). Transcriptional repression by sumoylation of Epstein–Barr virus BZLF1 protein correlates with association of histone deacetylase. *J. Biol. Chem.* 285 23925–23935 20516063 10.1074/jbc.M109.095356 PMC2911316

[B31] Murata T. Kondo Y. Sugimoto A. Kawashima D. Saito S. Isomura H. (2012). Epigenetic histone modification of Epstein–Barr virus BZLF1 promoter during latency and reactivation in Raji cells. *J. Virol.* 86 4752–4761 22357272 10.1128/JVI.06768-11 PMC3347330

[B32] Murata T. Noda C. Saito S. Kawashima D. Sugimoto A. Isomura H. (2011). Involvement of Jun dimerization protein 2 (JDP2) in the maintenance of Epstein–Barr virus latency. *J. Biol. Chem.* 286 22007–22016 21525011 10.1074/jbc.M110.199836 PMC3121345

[B33] Murata T. Sato Y. Nakayama S. Kudoh A. Iwahori S. Isomura H. (2009). TORC2, a coactivator of cAMP-response element-binding protein, promotes Epstein–Barr virus reactivation from latency through interaction with viral BZLF1 protein. *J. Biol. Chem.* 284 8033–8041 19164291 10.1074/jbc.M808466200 PMC2658097

[B34] Ramasubramanyan S. Osborn K. Flower K. Sinclair A. J. (2012). Dynamic chromatin environment of key lytic cycle regulatory regions of the Epstein–Barr virus genome. *J. Virol.* 86 1809–1819 22090141 10.1128/JVI.06334-11 PMC3264371

[B35] Ruf I. K. Rawlins D. R. (1995). Identification and characterization of ZIIBC, a complex formed by cellular factors and the ZII site of the Epstein–Barr virus BZLF1 promoter. *J. Virol.* 69 7648–7657 7494273 10.1128/jvi.69.12.7648-7657.1995 PMC189705

[B36] Speck S. H. Chatila T. Flemington E. (1997). Reactivation of Epstein–Barr virus: regulation and function of the BZLF1 gene. *Trends Microbiol.* 5 399–405 9351176 10.1016/S0966-842X(97)01129-3

[B37] Tan J. Yang X. Zhuang L. Jiang X. Chen W. Lee P. L. (2007). Pharmacologic disruption of polycomb-repressive complex 2-mediated gene repression selectively induces apoptosis in cancer cells. *Genes Dev.* 21 1050–1063 17437993 10.1101/gad.1524107 PMC1855231

[B38] Toth Z. Maglinte D. T. Lee S. H. Lee H. R. Wong L. Y. Brulois K. F. (2010). Epigenetic analysis of KSHV latent and lytic genomes. *PLoS Pathog.* 6:e1001013 10.1371/journal.ppat.1001013 PMC2908616 20661424

[B39] Tsurumi T. Fujita M Kudoh A. (2005). Latent and lytic Epstein–Barr virus replication strategies. *Rev. Med. Virol.* 15 3–15 15386591 10.1002/rmv.441

[B40] Wang F. Z. Roy D. Gershburg E. Whitehurst C. B. Dittmer D. P. Pagano J. S. (2009). Maribavir inhibits Epstein–Barr virus transcription in addition to viral DNA replication. *J. Virol.* 83 12108–12117 19759127 10.1128/JVI.01575-09 PMC2786727

[B41] Wang H. Zhao Y. Li L. McNutt M. A. Wu L. Lu S. (2008). An ATM- and Rad3-related (ATR) signaling pathway and a phosphorylation-acetylation cascade are involved in activation of p53/p21Waf1/Cip1 in response to 5-aza-2′-deoxycytidine treatment. *J. Biol. Chem.* 283 2564–2574 17977830 10.1074/jbc.M702454200

[B42] Wozniak R. J. Klimecki W. T. Lau S. S. Feinstein Y. Futscher B. W. (2007). 5-Aza-2′-deoxycytidine-mediated reductions in G9A histone methyltransferase and histone H3 K9 di-methylation levels are linked to tumor suppressor gene reactivation. *Oncogene* 26 77–90 16799634 10.1038/sj.onc.1209763

[B43] Yu X. Wang Z. Mertz J. E. (2007). ZEB1 regulates the latent-lytic switch in infection by Epstein–Barr virus. *PLoS Pathog.* 3:e194 10.1371/journal.ppat.0030194 PMC2134958 18085824

[B44] Zheng Z. Li L. Liu X. Wang D. Tu B. Wang L. (2012). 5-Aza-2′-deoxycytidine reactivates gene expression via degradation of pRb pocket proteins. *FASEB J.* 26 449–459 21990374 10.1096/fj.11-190025

